# Intestinal parasites in the Neolithic population who built Stonehenge (Durrington Walls, 2500 BCE)

**DOI:** 10.1017/S0031182022000476

**Published:** 2022-07

**Authors:** Piers D. Mitchell, Evilena Anastasiou, Helen L. Whelton, Ian D. Bull, Mike Parker Pearson, Lisa-Marie Shillito

**Affiliations:** 1 Department of Archaeology, University of Cambridge, Henry Wellcome Building, Cambridge CB2 1QH, UK; 2 Organic Geochemistry Unit, School of Chemistry, University of Bristol, Cantock's Close, Bristol BS8 1TS, UK; 3 Institute of Archaeology, UCL, 31-34 Gordon Square, London WC1H 0PY, UK; 4 School of History, Classics and Archaeology, Newcastle University, Newcastle upon Tyne NE1 7RU, UK

**Keywords:** Capillariasis, *Dibothriocephalus*, fish tapeworm, helminth, Neolithic, palaeoparasitology

## Abstract

Durrington Walls was a large Neolithic settlement in Britain dating around 2500 BCE, located very close to Stonehenge and likely to be the campsite where its builders lived during its main stage of construction. Nineteen coprolites recovered from a midden and associated pits at Durrington Walls were analysed for intestinal parasite eggs using digital light microscopy. Five (26%) contained helminth eggs, 1 with those of fish tapeworm (likely *Dibothriocephalus dendriticus*) and 4 with those of capillariid nematodes. Analyses of bile acid and sterol from these 5 coprolites show 1 to be of likely human origin and the other 4 to likely derive from dogs. The presence of fish tapeworm reveals that the Neolithic people who gathered to feast at Durrington Walls were at risk of infection from eating raw or undercooked freshwater fish. When the eggs of capillariids are found in the feces of humans or dogs it normally indicates that the internal organs (liver, lung or intestines) of animals with capillariasis have been eaten, and eggs passed through the gut without causing disease. Their presence in multiple coprolites provides new evidence that internal organs of animals were consumed. These novel findings improve our understanding of both parasitic infection and dietary habits associated with this key Neolithic ceremonial site.

## Graphical abstract

**Figure:**
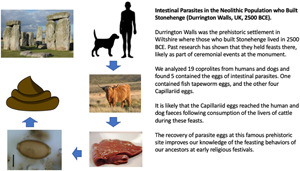

## Introduction

**Fig. 1.**
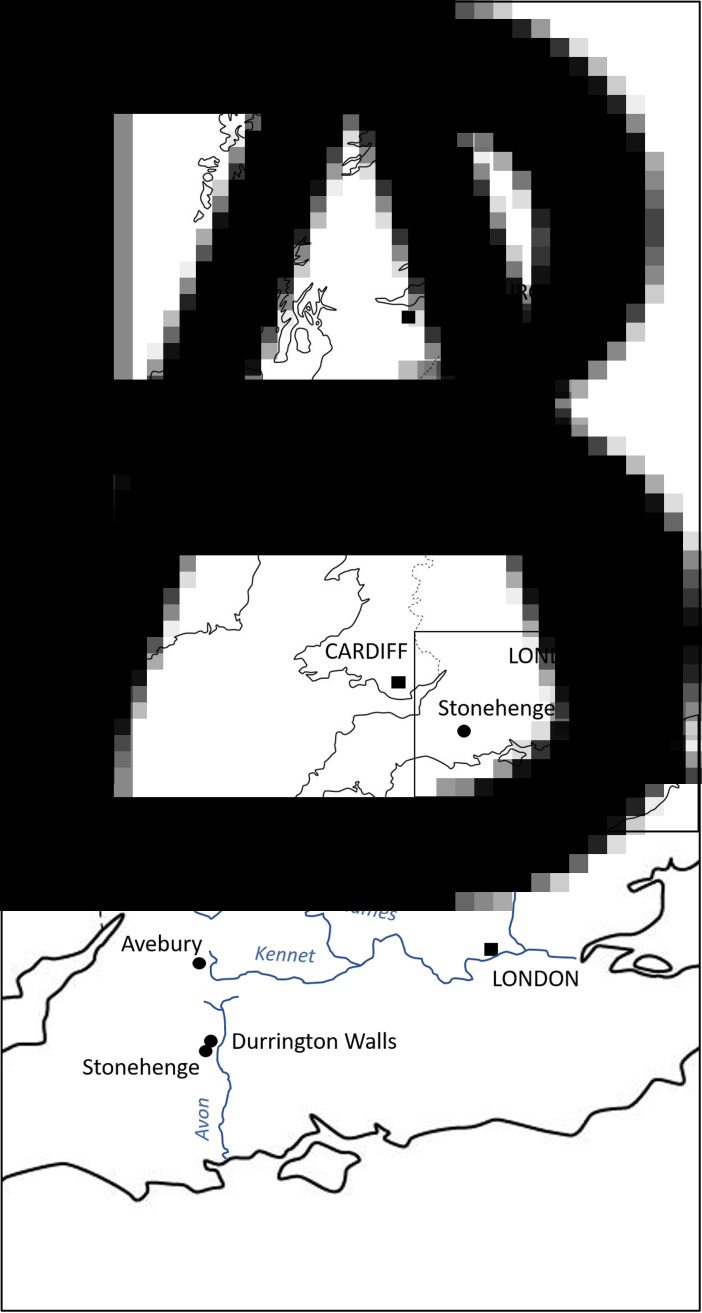
Map indicating location of the Durrington Walls and Stonehenge.

Durrington Walls is a large Neolithic henge located just 2.8 km from Stonehenge, Wiltshire (UK) (Fig. 1). Prior to its construction as a henge, it was the site of a large settlement estimated to start in 2535–2475 cal BCE (95% probability) and in use for 0–55 years (95% probability), possibly for no more than a decade (Parker Pearson *et al*., 2015: 51). Its period of occupation broadly coincides with the construction of Stonehenge's sarsen circle and trilithons, suggesting that this was the builders' village for Stonehenge stage 2 (Parker Pearson *et al*., 2007, 2020: 169–171). Analysis of animal bone and food residues in pottery from Durrington Walls shows that it was a place of feasting, especially during the winter months (Albarella and Serjeantson, 2002; Wright *et al*., 2014; Craig *et al*., 2015; Chan *et al*., 2016).

Only a very limited amount is known about parasite infection in the prehistoric population of Britain up to the Neolithic (Mitchell, 2013; Anastasiou, 2015). In the Mesolithic layers of peat at Goldcliff in South Wales (5840–5620 BCE) whipworm eggs were recovered, which may have come from humans (*Trichuris trichiura*) or pigs (*Trichuris suis*) (Dark, 2004). We have no data at all for parasites affecting people in Neolithic Britain. This is in contrast to the quite extensive research undertaken in the rest of Europe at the well preserved Neolithic alpine lakeside settlements in France, Germany and Switzerland (Bouchet *et al*., 1995; Dommelier *et al*., 1998; Le Bailly *et al*., 2005, 2007; Maicher *et al*., 2019), Spain (Maicher *et al*., 2017) and also Mesolithic Sweden (Bergman, 2018) and Ireland (Perri *et al*., 2018). Clearly research investigating parasites at Neolithic sites in Britain is needed.

The aim of this study is to investigate firstly whether those humans and animals living at the Neolithic settlement of Durrington Walls were infected by parasites, and secondly whether we can detect the eggs of non-infective parasites that help us to better understand the diet and food behaviours of the population. Since the Stonehenge environs are one of the most thoroughly investigated regions of prehistoric Britain, this contributes to a growing corpus of evidence and knowledge about Neolithic lifeways.

## Materials and methods

### Durrington Walls

The settlement of Durrington Walls (and the adjacent site of Woodhenge) is located 2.8 km northeast of Stonehenge. The 2 sites were formerly linked by the river Avon, from which one avenue leads to Stonehenge and the other to Durrington Walls (Parker Pearson *et al*., 2007, 2020: 409–497; French *et al*., 2012). Excavations outside the east entrance of Durrington Walls by the Stonehenge Riverside project in 2004–2007 uncovered 7 house floors, substantial midden deposits and over 100 pits. The main midden, shared by 6 of the houses, contained a large assemblage of Grooved Ware pottery, worked stone tools and over 38 000 animal bones, all associated with feasting activities. Some 90% of the bones come from pigs, with less than 10% from cattle. The majority of the pigs was killed at around 9 months of age, consistent with farrowing in spring and slaughter in winter (Wright *et al*., 2014). Isotopic analysis indicates the livestock were brought to the site from many different regions of southern Britain, providing a proxy for the catchment from which the people themselves derived (Viner *et al*., 2010; Evans *et al*., 2019; Madgwick *et al*., 2019, 2021).

**Fig. 2.**
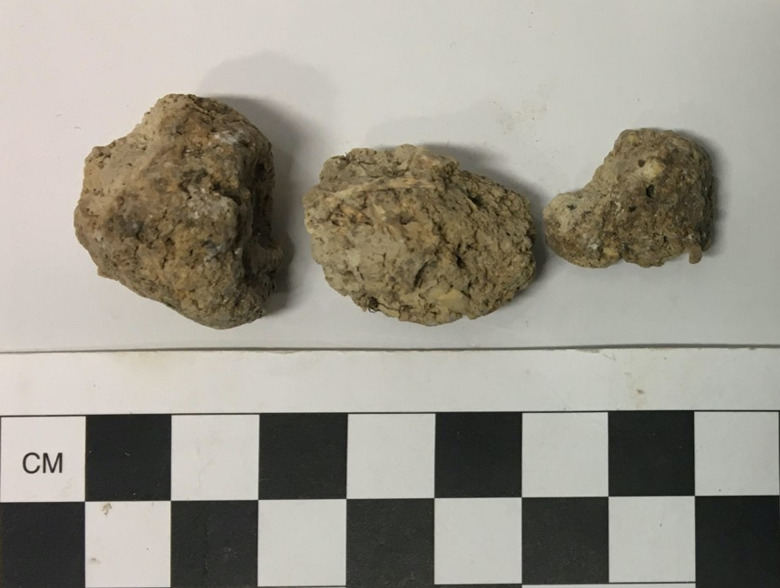
Fragments of the human coprolite DW11465.

Some of the pits contained coprolites (Fig. 2). These are partially mineralized ancient fecal material, the size and morphology of which indicate they likely come from omnivores and not herbivores. Identifying the species of coprolite on the basis of morphology is difficult, and this varies substantially depending on the age, diet and health of an individual (Shillito *et al*., 2020*a*). However, biomolecular methods can provide a more conclusive indication of species, and have been used to distinguish between different mammals at archaeological sites (Harrault *et al*., 2019; Romaniuk *et al*., 2020; Shillito *et al*., 2020*b*).

### Microscopy

Nineteen coprolites from the midden area were available for analysis. Sample preparation and analysis was undertaken in the Ancient Parasites Laboratory at the University of Cambridge using our routine methodology (Anastasiou and Mitchell, 2013). A 1 g subsample of each coprolite was disaggregated (made into a liquid suspension) by adding 5 mL 0.5% trisodium phosphate to the subsample. Material within the size range of interest was isolated from the disaggregate using stacked microsieves with mesh measuring 300, 160 and 20 *μ*m. The material trapped on the 20 *μ*m sieve was washed free of the mesh. This would contain any helminth eggs present, as the typical size range of eggs from helminths in northern Europe is 20–150 *μ*m (Garcia, 2016: 1233). The suspension was centrifuged at 4000 rpm (3100 ***g***) for 5 min, and the supernatant then removed. The remaining pellet was mixed with glycerol and the entire subsample was viewed on a digital light microscope (Olympus BX40F microscope with GXCAM-9 digital camera) at 400× magnification to visualize any preserved parasite eggs.

### Fecal biomarker analysis

**Fig. 3.**
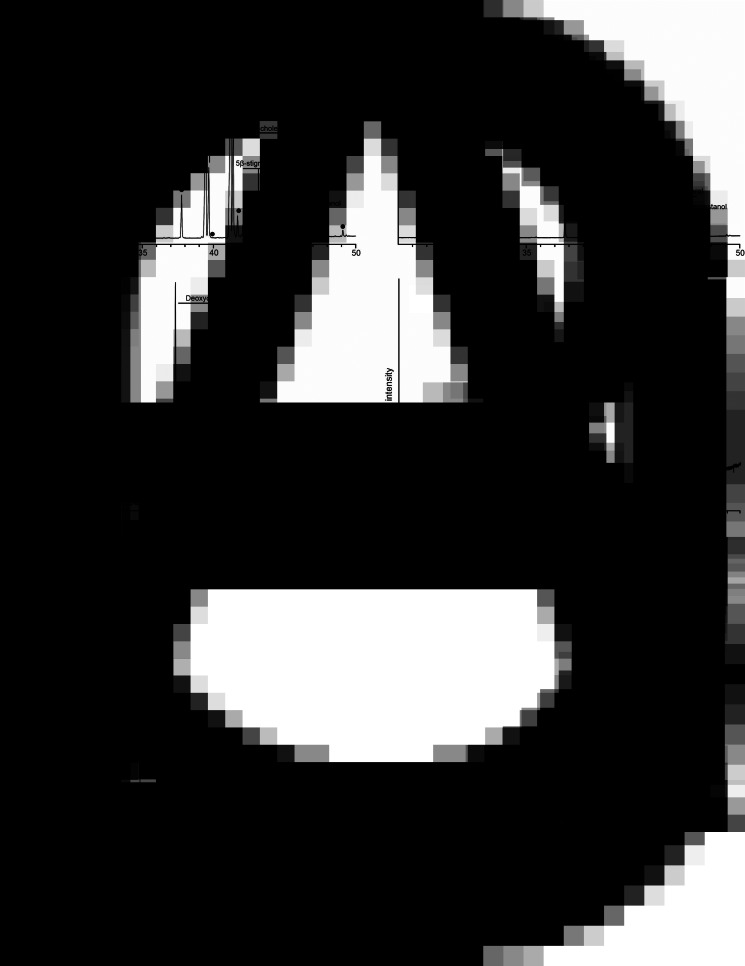
Partial gas chromatograms illustrating the distribution of steroid compounds in (A) human (DW11465) and (B) carnivore (DW082.1) coprolites, where ● denotes *n*-alcohols, and their corresponding bile acids in (C) human (DW11465) and (D) carnivore (DW082.1), where ■ denotes hydroxy fatty acid methyl esters (TMS derivatives) and ⧫ denotes *ω*-hydroxy fatty acid methyl esters (TMS derivatives). IS denotes added internal standards: Preg-5-en-3*β*-ol is the sterol standard and hyocholic acid is the standard for bile acids.

Those coprolites found to contain parasite eggs underwent further investigation to determine if the feces originated from humans or other animals that produce similar shaped feces, such as dogs. The use of fecal lipid biomarkers is a well-established method for determining coprolite species of origin (Bull *et al*., 2002; Shillito *et al*., 2011; Harrault *et al*., 2019; Shillito *et al*., 2020*b*) and identifying the presence of human and animal fecal waste in archaeological sediments (Brown *et al*., 2021). Approximately, 0.5 g of the coprolite was crushed using a mortar and pestle, then passed through a 2 mm sieve. Suitable amounts of two internal standards (hyocholic acid and preg-5-en-3*β*-ol; 50 *μ*L, 0.1 mg mL^−1^ solution) were added to the powdered samples. The lipids were then microwave extracted using an Ethos EX microwave-assisted extraction system [10 min ramp to 70°C (1000 W), 10 min hold at 70°C (1000 W) and 20 min cool down] using 10 mL of 2:1 DCM/MeOH (v/v). The total lipid extract (TLE) obtained was subsequently hydrolysed and the sterol and bile acid fractions were isolated as outlined in Elhmmali *et al*. (1997) and Bull *et al*. (1999). The fractions containing the target biomarkers were then analysed by gas chromatography and gas chromatography-mass spectrometry. Each of the coprolites yielded lipid profiles adequate for source identification. Four of the 5 coprolites analysed (DW248.614, DW082.1, DW248.616.1 and DW12164) were identified as likely dog in origin, and 1 coprolite was identified as likely human in origin (DW11465) (Fig. 3) by comparing with established values for each species (Wildgrube *et al*., 1986; Leeming *et al*., 1996; Elhmmali *et al*., 1997; Bull *et al*., 2002; Prost *et al*., 2017).

## Results

**Fig. 4.**
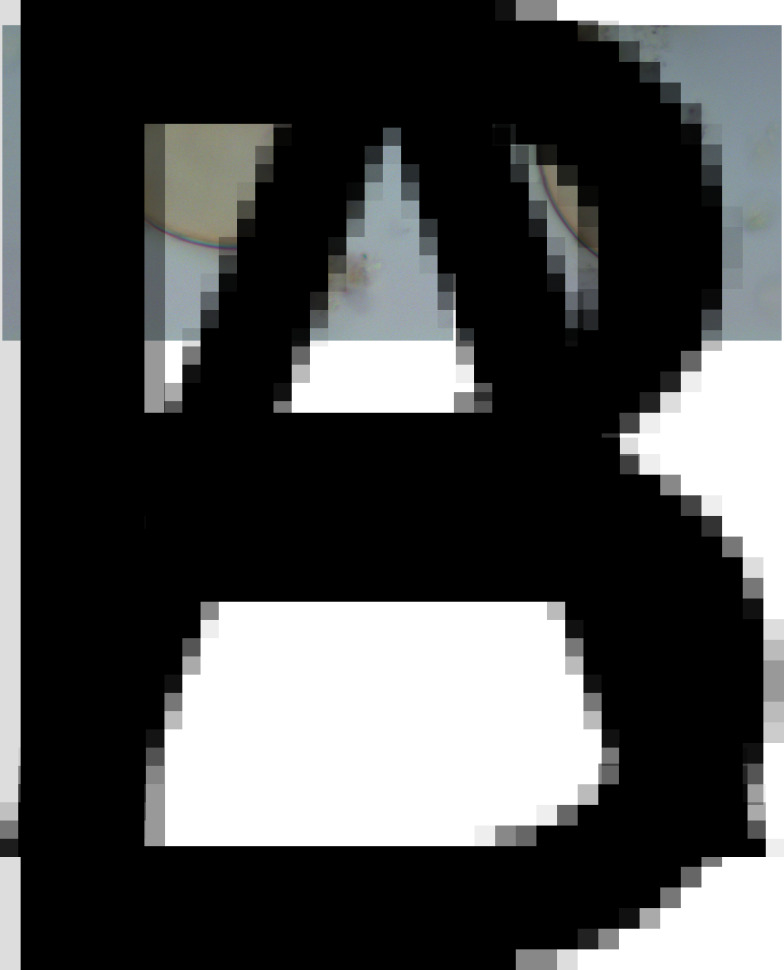
Fish tapeworm (likely *Dibothriocephalus dendriticus*) eggs from coprolite DW248.616.1 at Durrington Walls: (A) egg with operculum intact, dimensions 56 × 40 *μ*m^2^; (B) operculum lost, dimensions 56 × 42 *μ*m^2^. Scale bar indicates 20 *μ*m.

**Fig. 5.**
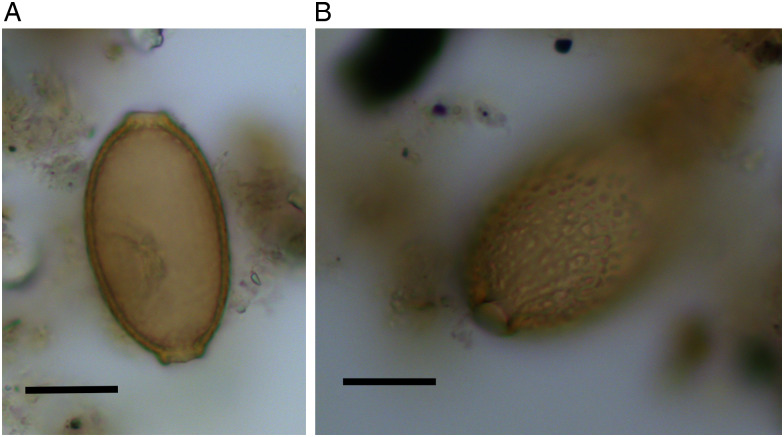
Capillariid egg from coprolite DW12164 at Durrington Walls. Images (A) and (B) are from the same egg; the surface structure of the egg is visible highlighting the network-like pattern on the surface (B). Scale bars indicate 20 *μ*m.

Five of the 19 coprolites contained parasite eggs. One likely dog coprolite (DW248.616.1) contained the eggs of fish tapeworm (Fig. 4). These each had a smooth opercular outline without opercular shoulders. Egg size ranged from 52 to 56 *μ*m × 35 to 42 *μ*m. These characteristics would be compatible with *Dibothriocephalus* sp. (also known as *Diphyllobothrium* sp.). Four other coprolites contained capillariid eggs, identified by their lemon-shape, network-like surface pattern, and dimensions of 50–56 *μ*m × 30–31 *μ*m (Fig. 5). One of these coprolites was of likely human origin (DW11465) and the other 3 from dogs (DW082.1, DW248.614 and DW12164). One coprolite contained 4 capillariid eggs, and the other 3 each contained 1 egg. The surface pattern was the same for all the capillariids identified, suggesting they were of the same species. Dimensions are detailed in Table 1.

**Table 1.** Details of the fish tapeworm and capillariid eggs found in the coprolites

Species	Egg count per g	Mean length (*μ*m) + s.d.	Mean width (*μ*m) + s.d.
*Dibothriocephalus dendriticus*	4	54.3 ± 1.8	39.3 ± 2.6
Capillariids	1–4	53.4 ± 2.2	30.4 ± 0.5

## Discussion

### Species identification

Today the species of fish tapeworm found in freshwater fish in northern Europe are *Dibothriocephalus dendriticus* and *Dibothriocephalus latus* (Chappell and Owen, 1969; Scholz *et al*., 2009; Brewster, 2016). Therefore it would be reasonable to investigate whether these eggs are compatible with either of those species. Research comparing the egg dimensions of different species of fish tapeworm has found that the typical size of *D. dendriticus* eggs range from 53 to 66 *μ*m in length to 38 to 45 *μ*m in width, while those of *D. latus* range from 60 to 81 *μ*m × 40 to 58 *μ*m (Leštinová *et al*., 2016). Since our eggs have dimensions of 52–56 *μ*m × 35–42 *μ*m, they would be most compatible with *D. dendriticus*. However, the ancient nature of these eggs means that over time their size might potentially have changed, so there will be a greater level of uncertainty regarding the species compared with if these had been modern samples.

A considerable number of parasitic helminths in the Capillariidae family is found in modern Europe. While dimensions and eggshell surface structure can help differentiate subgroups, few have eggs that possess sufficiently distinctive morphological appearance on the microscope for reliable diagnosis to the species level (Bouchet, 1997; Fugassa *et al*., 2008; Borba *et al*., 2019). They predominantly infect animals, but some may occasionally infect humans. Today the species most commonly found to infect humans is *Capillaria hepatica*. While infection results in eggs in the liver, it does not result in eggs being found in the human stool. If eggs of *C. hepatica* are found in the human stool it typically results from spurious infection (false parasitism), when raw or undercooked liver is eaten and the eggs contained are released following digestion of the liver tissue (Garcia, 2016: 209). Bearing in mind the surface network-like pattern and the dimensions, our images share some characteristics with the capillariid *Aonchotheca bovis*, which infects cattle (Justine and Ferte, 1988). Since we know that cattle were slaughtered and eaten at the site, it is plausible to suggest that the capillariid eggs found in 4 of the coprolites got there when humans and their dogs ate the internal organs of infected animals such as cattle in the preceding days.

### Prehistoric lifeways revealed through parasite remains

Here we have found evidence for parasite eggs in the preserved feces of humans and their dogs that gathered at Durrington Walls around 4500 years ago. Not only is this a key discovery due to the important nature of the site, but this is the earliest evidence for parasite infection in Britain where we can be confident of the species of the hosts. Previous research has found whipworm eggs (*Trichuris* sp.) in peat layers in South Wales dating to around 5840–5620 BC (Mesolithic period), but it is unknown whether they were from human or pig infection (Dark, 2004). In humans, roundworm (*Ascaris* sp.) and whipworm eggs were found at a Bronze Age farming settlement at Brean Down in Somerset (2100–600 BC) (Jones, 1990), while eggs of fish tapeworm (*D. dendriticus* and *D. latus*), *Echinostoma* sp., giant kidney worm (*Dioctophyma renale*), *Capillaria* sp. and probable pig whipworm (*T. suis*) were recovered from human coprolites at the marsh dwelling settlement at Must Farm in the fens of East Anglia (800–900 BC) (Ledger *et al*., 2019*b*). The contrasting species of parasite found at these two Bronze Age sites highlights how prehistoric lifestyle was key for explaining the profile of parasites that we might find in a population. This contrast has been found in Mesolithic and Neolithic sites in other regions of Europe as well, with populations living in lakeside villages often having more zoonotic species, and a broader range of species, than those who farmed crops and herded animals in dryer regions (Le Bailly *et al*., 2007; Anastasiou, 2015; Maicher *et al*., 2017; Anastasiou *et al*., 2018; Ledger and Mitchell, 2019). Over time we also see a gradual change from the Neolithic pattern of both zoonotic parasites and geohelminths such as roundworm and whipworm, to the later dominance of geohelminths and sometimes disappearance of zoonoses at some sites where they previously existed (Reinhard *et al*., 2013).

The presence of fish tapeworm eggs in 1 of the 19 coprolites suggests that the dog that deposited this piece of feces had previously eaten raw or undercooked freshwater fish and contracted fish tapeworm. This adds to our growing knowledge of parasites in Neolithic dogs (Tolar and Galik, 2019; Tolar *et al*., 2020; Maicher *et al*., 2021). While modern infection would lead to a high number of eggs in the feces, in ancient coprolites recovered from middens it is not uncommon for egg counts to be low, likely due to destruction of the eggs by fungi and insects over the centuries (see Ledger *et al*., 2019*a* for similar egg counts at a comparable Neolithic site). Since only 1 of the 19 coprolites contained fish tapeworm eggs, this would suggest either that infection was not common at the site, or that some helminth eggs originally present in other coprolites did not survive the 4500 years in the ground. This contrasts with the Bronze Age marshland settlement of Must Farm, where every human coprolite contained the eggs of either *Echinostoma* sp. or *Dibothriocephalus* sp., from eating raw fish and other aquatic animals (Ledger *et al*., 2019*b*). It is likely that preservation has played a role here; deposits at Durrington Walls are from dryland contexts in contrast to the exceptional preservation in the waterlogged deposits of Must Farm. Fish tapeworm has been found at a number of prehistoric European sites during the Mesolithic and Neolithic, and at that time is thought to reflect the component of the diet made up by hunting and gathering wild foods (Le Bailly and Bouchet, 2013).

We should consider whether this dog may have contracted the fish tapeworm infection while residing at Durrington Walls, or elsewhere. Since the settlement at Durrington Walls appears to have been occupied on a largely seasonal basis, predominantly in the winter periods, it is possible that the dog had arrived already infected with the parasite. At Durrington Walls no bones of freshwater fish were recovered, despite excellent preservation of faunal remains, and there was no evidence of freshwater fish oil in the pottery analysed. This would suggest that any consumption of fish by dogs could have occurred elsewhere, in the settlements scattered across southern Britain where the people who came to Durrington Walls in the winter would have spent the rest of the year. Stable isotope analysis of collagen extracted from the few human remains found at Durrington Walls (Craig *et al*., 2015) does not rule out the consumption of freshwater fish over several years prior to death, but if so this was likely to have been away from the site.

The capillariid eggs found in 1 human and 3 dog coprolites do not indicate infection, but rather reflects the consumption of the internal organs of animals. If the liver or lungs of animals were eaten without being thoroughly cooked, then the capillariid eggs they contained could have passed through the intestinal tract and end up in the feces due to spurious infection (false parasitism). These findings emphasize the importance of coprolites in providing evidence for consumption of items that are not visible through other lines of evidence (Blong *et al*., 2020; Shillito *et al*., 2020*a*). Earlier archaeological research on animal bone and pottery residues undertaken at Durrington Walls indicates that feasts were held at the site involving large numbers of people and animals who travelled to the area, likely part of seasonal gatherings and ceremonies associated with Stonehenge and its surrounding monuments (Craig *et al*., 2015; Evans *et al*., 2019; Madgwick *et al*., 2019, 2021), and show that principally pork and beef were consumed (Albarella and Serjeantson, 2002; Wright *et al*., 2014). While compound specific isotope analysis of C16:0 and C18:0 fatty acids in pottery can distinguish between carcases and dairy fats, they cannot distinguish fats from organs compared to other parts of the animal carcases. It is also possible that residues in pottery may relate to storage of materials for craft purposes rather than food preparation (Shillito, 2019). Coprolites on the other hand, contain items that were unequivocally ingested by an individual. It is possible that the capillariid eggs in these coprolites could have been consumed by the people living at Durrington Walls when they ate the internal organs of cattle during these feasts. The left overs may then have been fed to their dogs, explaining the capillariid eggs in their coprolites.

## Conclusion

Here we have investigated fecal remains of humans and dogs that gathered at the large settlement of Durrington Walls during the Late Neolithic period. Using bile acid and sterol analysis we were able to determine the likely species of origin of each coprolite that contained parasite eggs. One coprolite from a dog contained the eggs of fish tapeworm, indicating it had likely eaten raw or undercooked fish and contracted the parasite. This is intriguing as there is very little other evidence of fishing (marine or fresh water) during the British Late Neolithic period. Four coprolites contained the eggs of Capillariidae nematodes, most probably indicating that both humans and dogs had eaten the internal organs of animals infected by this parasite. These results provide a new line of evidence for the consumption of animal organs, which has not been possible using more traditional forms of archaeological evidence.
